# Causal effects of denture wearing on epigenetic age acceleration and the mediating pathways: a mendelian randomization study

**DOI:** 10.1186/s12903-024-04578-y

**Published:** 2024-07-13

**Authors:** Xin Chen, Zheng Cheng, Junyu Xu, Qianyi Wang, Zhibai Zhao, Qianglin Jiang

**Affiliations:** 1 Department of Oral and Maxillofacial Surgery, Jiangyin People’s Hospital Affiliated to Nantong University, No.163, Shoushan Road, Jiangyin, 214400 Jiangsu Province China; 2 Department of Cardiology, Jiangyin People’s Hospital Affiliated to Nantong University, No.163, Shoushan Road, Jiangyin, 214400 Jiangsu Province China; 3 Department of Oral Mucosal Diseases, The Affiliated Stomatological Hospital of Nanjing Medical University, Nanjing, China

**Keywords:** Epigenetic age acceleration, Dentures, Mendelian randomization analysis, Oral health, Mediation analysis

## Abstract

**Background:**

The epigenetic-age acceleration (EAA) represents the difference between chronological age and epigenetic age, reflecting accelerated biological aging. Observational studies suggested that oral disorders may impact DNA methylation patterns and aging, but their causal relationship remains largely unexplored. This study aimed to investigate potential causal associations between dental traits and EAA, as well as to identify possible mediators.

**Methods:**

Using summary statistics of genome-wide association studies of predominantly European ancestry, we conducted univariable and multivariable Mendelian randomization (MR) to estimate the overall and independent effects of ten dental traits (dentures, bleeding gums, painful gums, loose teeth, toothache, ulcers, periodontitis, number of teeth, and two measures of caries) on four EAA subtypes (GrimAge acceleration [GrimAA], PhenoAge acceleration [PhenoAA], HannumAge acceleration [HannumAA] and intrinsic EAA [IEAA]), and used two-step Mendelian randomization to evaluate twelve potential mediators of the associations. Comprehensive sensitivity analyses were used to verity the robustness, heterogeneity, and pleiotropy.

**Results:**

Univariable inverse variance weighted MR analyses revealed a causal effect of dentures on greater GrimAA (β: 2.47, 95% CI: 0.93–4.01, *p =* 0.002), PhenoAA (β: 3.00, 95% CI: 1.15–4.85, *p =* 0.001), and HannumAA (β: 1.96, 95% CI: 0.58–3.33, *p =* 0.005). In multivariable MR, the associations remained significant after adjusting for periodontitis, caries, number of teeth and bleeding gums. Three out of 12 aging risk factors were identified as mediators of the association between dentures and EAA, including body mass index, body fat percentage, and waist circumference. No evidence for reverse causality and pleiotropy were detected (*p* > 0.05).

**Conclusions:**

Our findings supported the causal effects of genetic liability for denture wearing on epigenetic aging, with partial mediation by obesity. More attention should be paid to the obesity-monitoring and management for slowing EAA among denture wearers.

**Supplementary Information:**

The online version contains supplementary material available at 10.1186/s12903-024-04578-y.

## Background

Aging is characterized by a gradual decline in numerous biological functions over time, contributing to increased susceptibility to age-related illnesses and mortality [1]. While chronological aging remains consistent, biological aging varies widely and can be influenced by genetics, environmental exposures, and health behaviors. Among potential indicators of biological age, the epigenetic clock, which measures DNA methylation at multiple cytosine-phosphate-guanine sites, is considered particularly reliable [2]. Epigenetic-age acceleration (EAA) quantifies the difference between chronological age and epigenetic age, indicating accelerated biological aging [3]. Identifying genetic and environmental factors influencing these measures within populations is a critical research goal.

Oral disorders, including periodontitis, denture wearing, and caries, significantly impact aging and related dysfunctions [4]. For example, individuals with moderate to severe periodontitis exhibit a 47% higher likelihood of having shorter telomeres compared to those with mild or no periodontitis, as observed in the NHANES 1999–2002 study [5]. Multiple observational studies supported periodontitis as a risk factor for accelerated aging [6, 7]. However, research on other dental traits, such as gingivitis and ulcers, and their impact on epigenetic age remains limited. Theoretically, proinflammatory environments accelerate telomere shortening due to oxidative stress and increased leukocyte mitosis rates [8]. Poor oral health could exacerbate inflammation and oxidative stress, potentially influencing EAA. Furthermore, individuals with poor oral health may avoid challenging-to-chew foods like resh fruits, vegetables, and whole grains, leading to deficiencies in essential vitamins and trace elements (e.g., Vitamin B12, iron), contributing to frailty and EAA [9, 10]. Additionally, compromised oral health weakens immune defenses, heightening susceptible to systemic illnesses that affect overall biological function [11].

Based on these theoretical models, we hypothesize that poorer oral health would be related with accelerated biological aging. However, aging itself compromises bone mineral density, while factors like reduced salivary flow and fewer dental visits increase susceptibility to periodontitis and caries in old adults [12]. Importantly, existing studies are predominantly based on small sample sizes, potentially introducing bias from extensive confounders and susceptibility to reverse causation [6, 12]. Thus, whether specific dental factors causally contribute to EAA remains unclear and warrants systematic investigation.

Mendelian randomization (MR) offers a robust approach to establish causal relationships by utilizing genetic variations directly linked to exposures [13]. The random distribution of genetic variants during conception mimics randomized controlled trials, reducing bias in causal inferences related to age and sex [14]. Moreover, since genotypes precede disease onset and are generally unaffected by disease progression, the risk of reverse causality is minimized [3]. Recent MR studies have strongly supported the causal link between dentures and conditions like coronary artery disease, heart failure, and earlier age at parents’ death [15, 16]. Multivariable MR (MVMR) further refines understanding by estimating complex pathways through which exposures influence outcomes, enhancing causal inference in mediating effects [17]. Recognizing the structure of the potential oral-aging relationship is of particular importance, considering that modifying tooth count or alveolar bone mass is challenging once the loss occurs.

In this study, we conducted two-sample univariable MR (UVMR) and MVMR analyses to investigate causal associations between dental traits and EAA. We specifically investigated mediating effects of modifiable risk factors on EAA development, aiming to enhance clinical relevance.

## Methods

### Study design

**Fig. 1:**
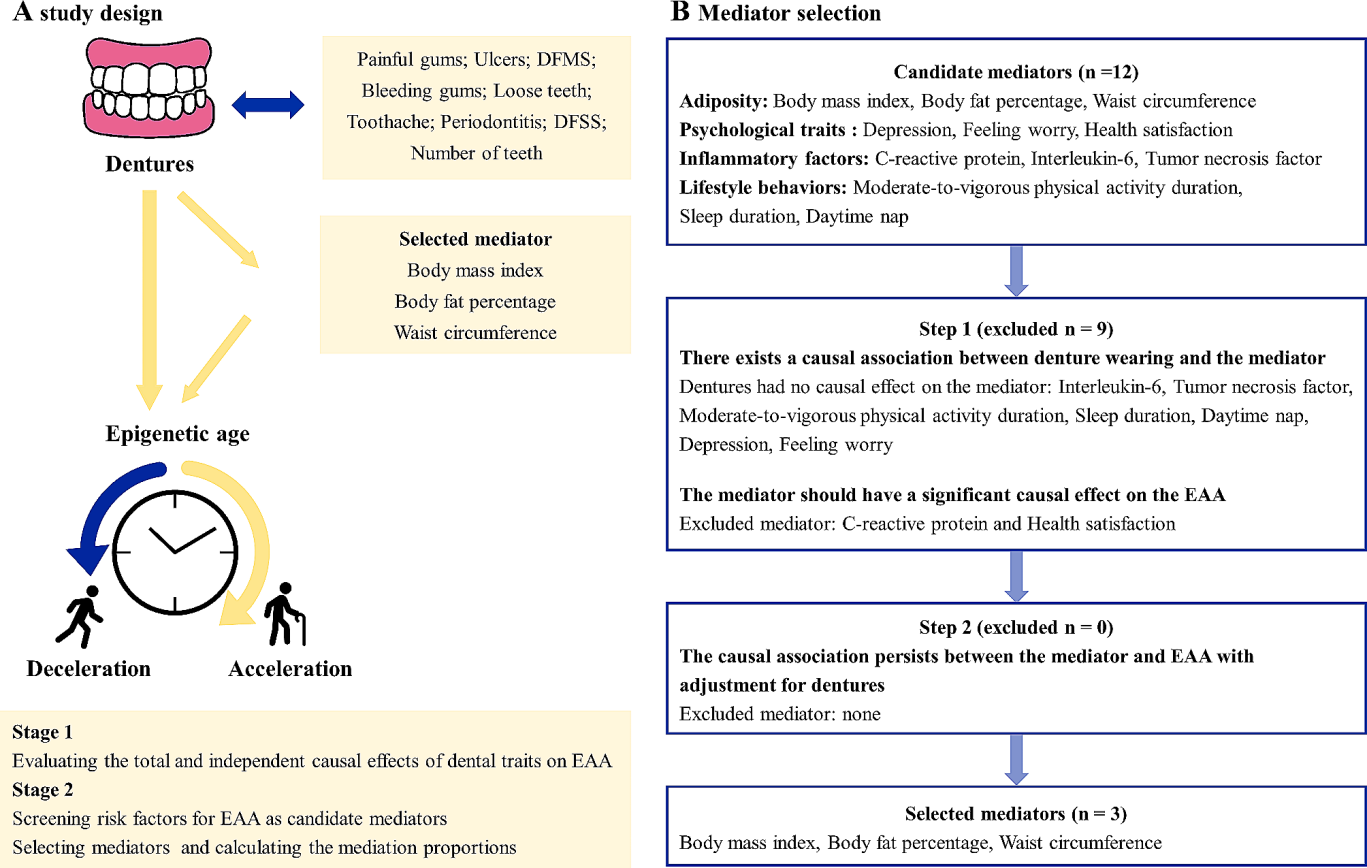
Overview of the study design. (**A**) Study design; (**B**) Mediator selection process in phase 2. This study consisted of 2 stages of analyses. In stage 1, we assessed the total and independent causal associations of ten dental traits with epigenetic-age acceleration (EAA) using univariable Mendelian randomization (UVMR) and multivariable Mendelian randomization (MVMR). Both UVMR and MVMR suggested that only dentures had a causal effect on increased EAA. In stage 2, we first screened candidate risk factors for EAA, and then identify potential causal mediators in the associations of dentures with EAA using two-step MR. DMFS, the sum of decayed, missing, and filled tooth surfaces; DFSS, the sum of decayed and filled tooth surface per available tooth surface; Nteeth, number of teeth

An overview of the study design is presented in Fig. 1. Initially, we meticulously examined the causal associations of ten dental traits with four subtypes of EAA through the application of univariable mendelian randomization (UVMR) and MVMR methodologies. Substantial causal evidence emerged, establishing a robust link between dentures and GrimAge acceleration (GrimAA), PhenoAge acceleration (PhenoAA), as well as HannumAge acceleration (HannumAA). We then summarized pivotal and shared risk factors believed to play a role in the pathways from oral health to EAA, aligning with the literature reviews [1, 14]. Twelve factors with available genome-wide association studies (GWASs) were thoughtfully selected as candidate mediators, and integrated into the mediation MR analysis using a two-step MR approach. To ensure the validity of causal effects, MR design must fulfill three key assumptions: [1] genetic instruments must be strongly associated with the exposure; [2] genetic instruments are independent of confounders associated with both the exposure and outcome; [3] genetic variants could only influence the outcomes through the exposure [18]. In addition, this study adheres to the guidelines set forth in the Strengthening the Reporting of Observational Studies in Epidemiology Using Mendelian Randomization guideline [13].

### Data sources for dental traits

A total of 10 dental traits, comprising clinical metrics of dental conditions from the Gene-Lifestyle Interactions in Dental Endpoints (GLIDE) consortium and self-reported oral health information from the UK Biobank, were carefully selected [16]. Trained professionals assessed each clinical trait using dental records, which included measures of caries experience (the sum of decayed, missing, and filled tooth surfaces, DMFS, *N* = 26,792) and the sum of decayed and filled tooth surface per available tooth surface (DFSS, *N* = 26,533), number of teeth (Nteeth, *N* = 27,949), and the dichotomous classification of periodontal health and disease (17,353 cases, 28,210 controls). Self-reported oral health was evaluated through a multiple-choice question: ‘Do you have any of the following? (You can select more than one answer)’. Possible answers included ‘Dentures’, ‘Bleeding gums’, ‘Painful gums’, ‘Loose teeth’, ‘Toothache’, or ‘Ulcers’. Participants selecting one of these answers were coded as cases, while those who did not select were coded as reference. The number of cases and controls for each trait were as follows: dentures (77,732 cases, 383,381 controls), bleeding gums (60,218 cases, 400,895 controls), loose teeth (18,981 cases, 442,132 controls), toothache (18,964 cases, 442,149 controls), painful gums (13,314 cases, 447,799 controls), and ulcers (47,102 cases, 414,011 controls). The abbreviations used in the text is listed in Table 1 and detailed information on all GWAS was shown in Supplementary Table 1.

**Table 1:** The list of abbreviations used in the text

Abbreviation	Definition
EAA	Epigenetic-age acceleration
COPD	Chronic obstructive pulmonary disease
MR	Mendelian randomization
MVMR	Multivariable mendelian randomization
UVMR	Univariable mendelian randomization
GWASs	Genome-wide association studies
GrimAA	GrimAge acceleration
PhenoAA	PhenoAge acceleration
HannumAA	HannumAge acceleration
IEAA	Intrinsic epigenetic-age acceleration
DMFS	The sum of decayed, missing, and filled tooth surfaces
DFSS	The sum of decayed and filled tooth surface per available tooth surface
Nteeth	Number of teeth
BMI	Body mass index
IL-6	Interleukin-6
TNF	Tumor necrosis factor
SNPs	Single nucleotide polymorphisms
IVW	Inverse variance weighted
MV-IVW	Multivariable inverse variance weighted
MR-PRESSO	Mendelian randomized polymorphism RESidual Sum and Outlier

### Data sources for EAA

Several metrics of EAA have been formulated, each capturing distinct facets of the aging. These included intrinsic EAA (IEAA), depicting aging regardless of blood cell-type composition [19], and HannumAA, which is more indicative of extrinsic aging [20]. The second-generation indicators of epigenetic-age acceleration, specifically GrimAA and PhenoAA, have advanced to incorporate aging-related traits and excel in predicting mortality and age-related cardiometabolic morbidities [21, 22]. The meta-analysis samples with DNA methylation GWAS data were from 28 cohorts involving 34,710 European participants [3]. Specifically, GrimAA (*N* = 34,467) and PhenoAA (*N* = 34,463) estimates were calculated using the Horvath Epigenetic Age Calculator or stand-along scripts. Additionally, we incorporated HannumAA (*N* = 34,449) and IEAA (*N* = 34,461) from the first representative epigenetic aging clocks, namely Hannum dataset and Intrinsic Horvath age, for side-by-side comparisons. More detailed information on data preparation for GWAS can be found from the original articles.

### Data sources for mediators

We selected 12 common modifiable factors, encompassing adiposity traits (body mass index [BMI], body fat percentage, waist circumference), psychology-related traits (depression, feeling worry, and health satisfaction), inflammatory factors (C-reactive protein [CRP], interleukin-6 [IL-6], Tumor necrosis factor [TNF]), and related lifestyle behaviors (moderate-to-vigorous physical activity duration, sleep duration, daytime nap) [1, 14]. It is essential to highlight that all GWAS data included in this study were explicitly centered on European populations, conducted with proper ethical approval and participant consent.

### Instruments selection

To identify suitable instrumental variables (IVs), a variety of quality control procedures were conducted [23]. Initially, we identified single nucleotide polymorphisms (SNPs) significantly linked to the exposure (*p* < 5 × 10^− 8^). IVs were then clumped, retaining independent SNPs (r^2^ ≥ 0.001, clumping window ≤ 10,000 kb) with the lowest p-values. Subsequently, the PhenoScanner V2 website (http://www.phenoscanner.medschl.cam.ac.uk) was utilized to exclude SNPs associated with potential confounders such as smoking, alcohol consumption, and diabetes, as well as the outcome of interest. Finally, R^2^ and F statistics were calculated as previously described [24], with an F statistic > 10 typically considered indicative of strong instrument strength.

### Statistical analysis

#### UVMR and MVMR analyses

We employed the inverse-variance weighted (IVW) method as the primary analysis in UVMR to ascertain causal estimates for the associations of each dental trait with EAA. To address the issue of multiple testing, a false discovery rate (FDR) method was applied, with an FDR q value of 0.05 serving as the threshold to define the evidence of a causal effect [14]. In addition, multivariable IVW (MV-IVW) analyses were conducted to assess whether the causal effects of specific influential factors on EAA remained independent of other exposure factors. Considering both the effect size and significance of causal associations, exposure factors with β coefficients > 0.5 and *p* < 0.05 in the same analyses were selected as covariates in the adjustment models. Moreover, bidirectional MR analyses were performed to investigate potential reverse causality from EAA to dental health. We considered IVW estimates indicative of causal associations only if they showed consistent direction and statistical significance in at least one sensitivity analysis and demonstrated no signs of pleiotropy.

#### Mediation MR analyses

We conducted a two-step MR analysis to assess the intermediary effects of risk factors between the influential dental factor and EAA [25]. Firstly, utilizing the UVMR approach, we calculated the causal impact of the selected dental exposure on the mediator (*β*1). Subsequently, employing the MVMR method, we estimated the causal effect of the mediator on EAA with adjustment for the specific dental factor (*β*2). The indirect effect was determined by multiplying the results from the two steps (*β*1 × *β*2) and dividing by the total effect. To establish the 95% confidence intervals for the mediation proportions, we applied the Delta method. The negative mediation proportion was constrained to a minimum threshold of 0% to signify the presence of a mediating effect.

To identify potential mediators, we adhered to the following criteria: [1] establishing a causal relationship between the dental factor and the mediator, as well as between the mediator and EAA; [2] ensuring that regardless of the adjustment for the selected dental trait, the causality between the mediator and EAA remained consistent.

#### MR sensitivity analysis

We performed sensitivity analyses, including MR Egger, weighted median, and Mendelian randomized polymorphism RESidual Sum and Outlier (MR-PRESSO) method, to confirm the robustness of the IVW results in the UVMR analysis. Additionally, we applied MVMR-Egger, MVMR-median, and MVMR-Lasso analyses to confirm the reliability of the MV-IVW results in the MVMR analysis. Heterogeneity among the instruments was assessed using Cochran’s Q test. The MR-Egger intercept test and the F-statistics were performed to examine the horizontal pleiotropy and instrument strength, respectively.

All analyses were performed using R (version 4.3.0) with the TwoSampleMR package (version 0.5.6), fdrtool (version 1.2.17), MRPRESSO (version 1.0), and MendelianRandomization (version 0.7.0).

## Results

### Effects of dental traits on EAA and the reverse effects

In the forward MR analysis, we adjusted the association threshold to 5 × 10^− 6^ due to the limited number of SNPs reaching previous genome-wide significance. Detailed SNPs following the initial screening process, elimination of confounders (such as smoking, alcohol consumption and diabetes), and data harmonization, can be found in Supplementary Tables 2–4. The F-statistics of IVs ranged from 20.9 to 1047.2, indicating no evidence of weak instrument bias.

**Fig. 2:**
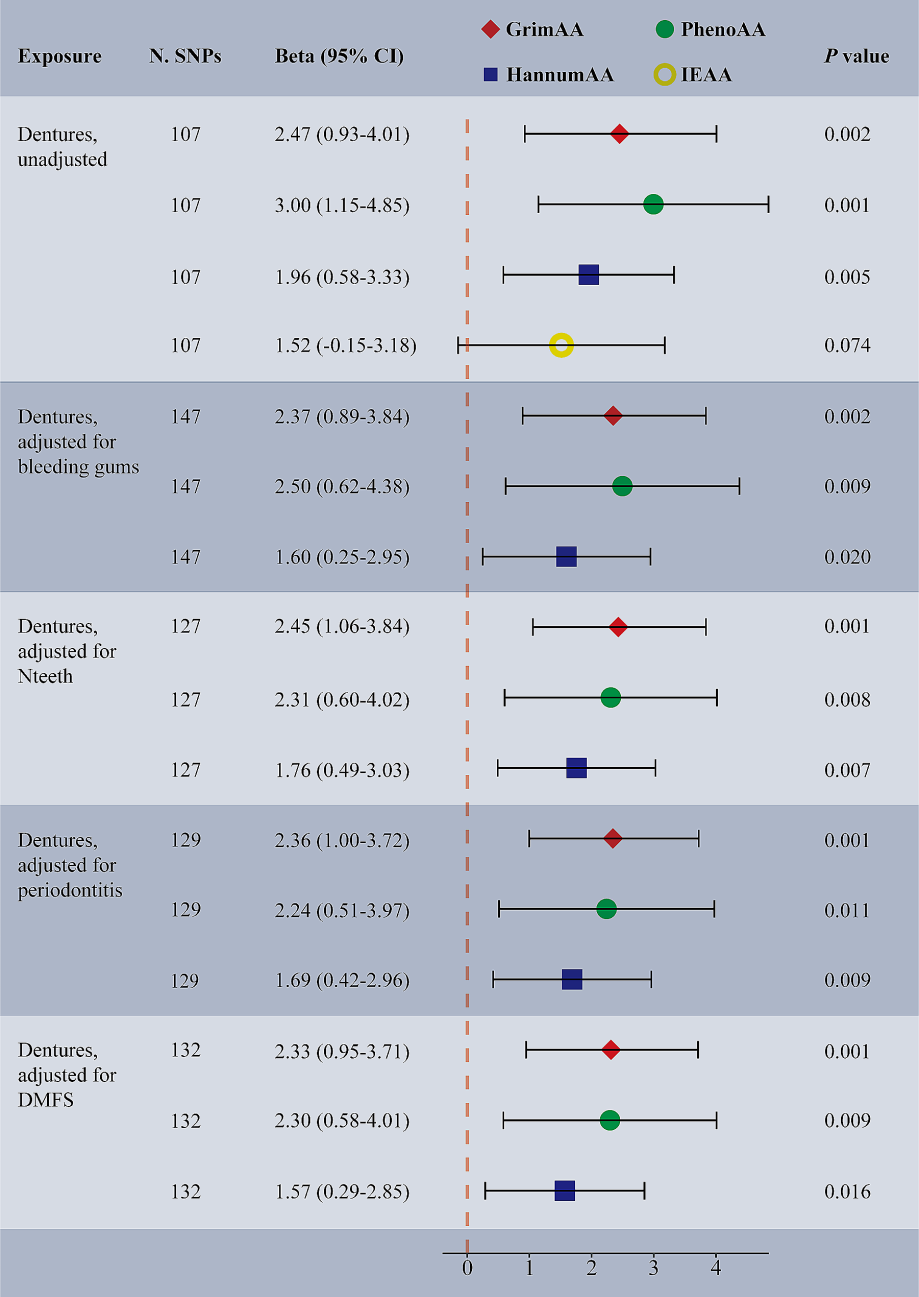
UVMR and MVMR estimates for the causal association between dentures and epigenetic-age acceleration. Beta (95% CI) were based on the IVW (UVMR) and MV-IVW (MVMR) analyses. CI, confidence interval; IVW, inverse variance weighted; MV-IVW, multivariable inverse variance weighted; UVMR, univariable Mendelian randomization; MVMR, multivariable Mendelian randomization; N. SNPs, number of SNPs used in MR. GrimAA, GrimAge acceleration; PhenoAA, PhenoAge acceleration; HannumAA, HannumAge acceleration; IEAA, intrinsic epigenetic-age acceleration

Among the phenotypes examined, only dentures exhibited strong causal associations with increased GrimAA (IVW β: 2.47, 95% CI: 0.93–4.01, *p =* 0.002, *q* = 0.022), PhenoAA (IVW β: 3.00, 95% CI: 1.15–4.85, *p =* 0.001, *q* = 0.005) and HannumAA (IVW β: 1.96, 95% CI: 0.58–3.33, *p =* 0.005, *q* = 0.023) after FDR adjustment for multiple comparisons (Fig. 2; Supplementary Table 5). These associations remained significant across the MRPRESSO method without notable outliers. Genetically proxied bleeding gums displayed a dominant yet nonsignificant association with IEAA (IVW β: 3.97, 95% CI: 0.37–7.58, *p =* 0.031, *q* = 0.083). Conversely, the weighted median analysis uncovered a potential protective effect of Nteeth on smaller HannAA (β: -0.85, 95% CI: -1.57, -0.12, *p =* 0.022, *q* = 0.178). No significant or potential association was noted between other dental factors and EAA. The Cochran’s Q test showed possible heterogeneity for bleeding gums as an exposure but not for dentures and Nteeth. Simultaneously, we identified limited evidence of pleiotropy influencing these MR results.

As primary causes of denture use, caries and periodontitis were also selected as covariates in the MVMR analyses. Surprisingly, the positive association between dentures and GrimAA, PhenoAA, as well as HannumAA, persisted significant with adjustments for bleeding gums, Nteeth, periodontitis, or DMFS (Fig. 2; Supplementary Table 6). All MV-IVW estimates garnered support from at least one sensitivity method. The instrumental validity test indicated no evidence of weak instrument bias (all F statistics ≥ 23.15). Causal effects for dentures on PhenoAA exhibit heterogeneity, but no evidence of pleiotropy emerged.

In the reversed MR analysis, a total of 4 SNPs for GrimAA, 9 SNPs for HannumAA, 11 SNPs for PhenoAA, and 24 SNPs for IEAA were extracted, with F-statistic values ranging from 30.8 to 239.7 (Supplementary Table 7). The UVMR analysis indicated that none of the EAA measures had a causal effect on any dental trait (IVW all *p* ≥ 0.40, *q* ≥ 0.192) (Supplementary Table 8). Consistent results were observed using the weight median method and MR-Egger method. Additionally, the MR-Egger analyses provided minimal evidence of pleiotropy (*p* ≥ 0.151), except for the effect of PhenoAA on toothache (intercept = -0.001, *p* = 0.021) (Supplementary Table 8).

### Effect of dentures on mediators

In UVMR analysis, genetically predicted denture wearing exhibited a significant association with increased BMI (IVW β: 0.57, 95% CI: 0.25–0.88, *p <* 0.001), body fat percentage (IVW β: 0.50, 95% CI: 0.30–0.70, *p <* 0.001), waist circumference (IVW β: 0.51, 95% CI: 0.26–0.76, *p <* 0.001), CRP (IVW β: 0.47, 95% CI: 0.19–0.76, *p =* 0.001), and health satisfaction (IVW β: 0.33, 95% CI: 0.16–0.50, *p <* 0.001). Each IVW estimate was corroborated by at least one sensitivity analysis, ensuring robustness and reliability (Table 2). Genetic IVs of dentures demonstrated persistent heterogeneity but exhibited no pleiotropy with the selected mediators. Notably, seven other mediators were excluded as they were unaffected by dentures (Supplementary Table 9).

**Table 2:** UVMR estimating the causal associations between dentures and the selected mediators

Mediator	Method	N. SNPs	β (95% CI)	*P*		Cochran Q test					MR-Egger	
						Q statistic	*I* ^2^	*P*			Intercept	*P*
Body mass index	IVW	122	0.567 (0.250, 0.884)	< 0.001		1897.134	0.936	< 0.001			0.003	0.307
	MR-Egger	122	0.081 (-0.900, 1.063)	0.872								
	Weighted median	122	0.033 (-0.122, 0.189)	0.674								
	MR-PRESSO	95	0.373 (0.240, 0.505)	< 0.001								
Body fat percentage	IVW	122	0.503 (0.301, 0.704)	< 0.001		1204.709	0.900	< 0.001			0.001	0.723
	MR-Egger	122	0.395 (-0.232, 1.022)	0.219								
	Weighted median	122	0.034 (-0.095, 0.163)	0.604								
	MR-PRESSO	103	0.334 (0.226, 0.443)	< 0.001								
Waist circumference	IVW	122	0.509 (0.260, 0.759)	< 0.001		1268.679	0.905	< 0.001			0.000	0.877
	MR-Egger	122	0.451 (-0.326, 1.228)	0.257								
	Weighted median	122	0.082 (-0.065, 0.230)	0.275								
	MR-PRESSO	105	0.243 (0.117, 0.369)	< 0.001								
C-reactive protein	IVW	116	0.474 (0.191, 0.758)	0.001		161.918	0.290	0.003			-0.003	0.182
	MR-Egger	116	1.081 (0.151, 2.011)	0.025								
	Weighted median	116	0.497 (0.087, 0.908)	0.018								
	MR-PRESSO	115	0.426 (0.175, 0.678)	0.001								
Health satisfaction	IVW	122	0.331 (0.160, 0.501)	< 0.001		206.263	0.413	< 0.001			0.002	0.156
	MR-Egger	122	-0.033 (-0.560, 0.495)	0.903								
	Weighted median	122	0.230 (0.014, 0.445)	0.037								
	MR-PRESSO	121	0.297 (0.142, 0.452)	< 0.001								

Abbreviations: UVMR, univariable Mendelian randomization; N. SNPs, number of SNPs used in MR; CI, confidence intervals; IVW, inverse-variance weighted; PRESSO, pleiotropy residual sum and outlier

### Effects of mediators on EAA without and with adjustment for dentures

**Fig. 3:**
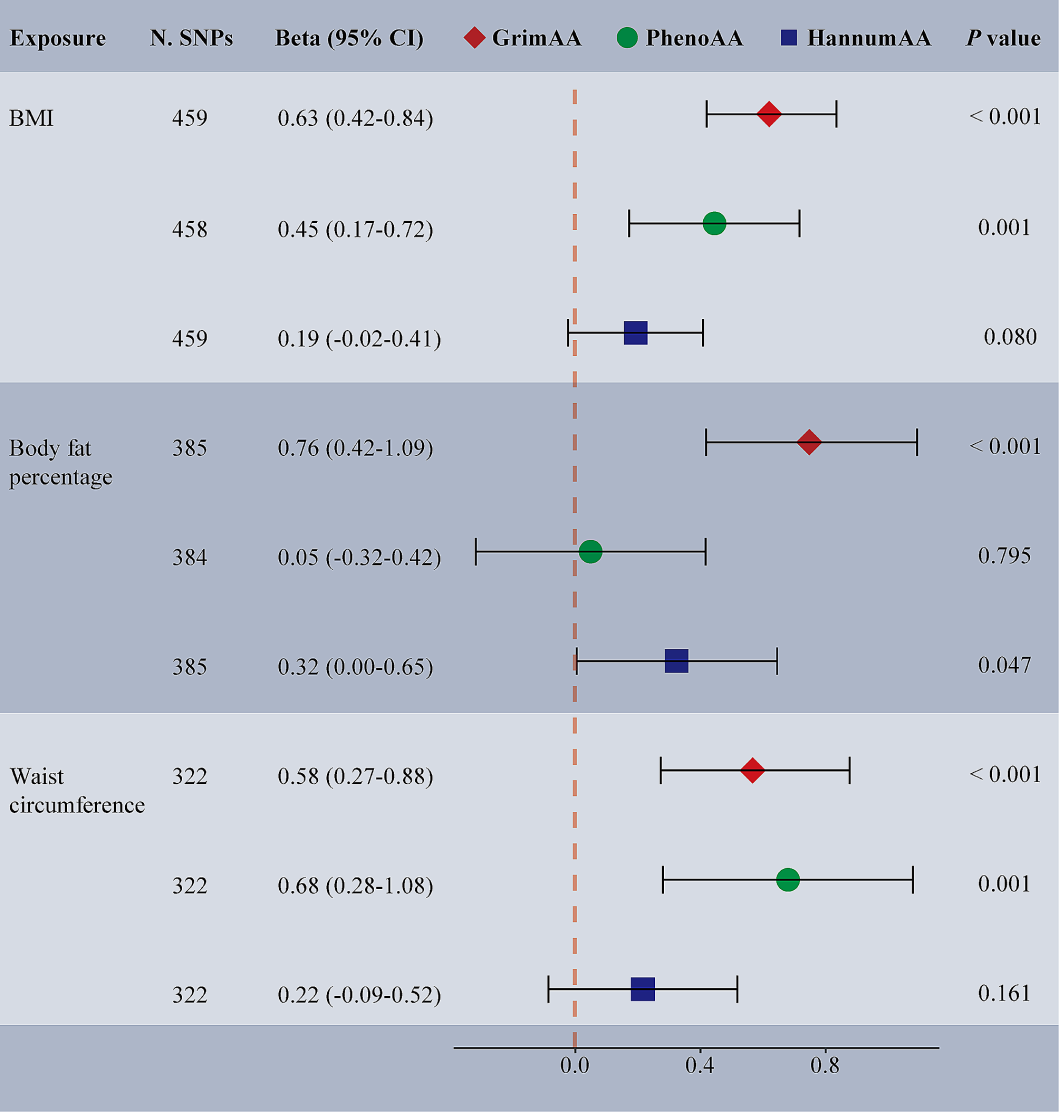
MVMR estimating the association of the selected mediator with epigenetic-age acceleration after adjusting for dentures. Beta (95% CI) were based on the MV-IVW (MVMR) analyses. CI, confidence intervals; MV-IVW, multivariable inverse-variance weighted; MVMR, multivariable Mendelian randomization; N. SNPs, number of SNPs used in MR; BMI, body mass index; GrimAA, GrimAge acceleration; PhenoAA, PhenoAge acceleration; HannumAA, HannumAge acceleration

UVMR results demonstrated that each genetically predicted adiposity trait was associated with greater GrimAA, PhenoAA, and HannumAA (IVW *p* < 0.05), with at least two sensitivity analyses confirming the IVW results (Supplementary Table 10). According to the MVMR analyses adjusting for dentures, there was still significant evidence of associations of BMI with GrimAA (MV-IVW β: 0.63, 95% CI: 0.42–0.84, *p <* 0.001), and PhenoAA (MV-IVW β: 0.45, 95% CI: 0.17–0.72, *p =* 0.001), as well as waist circumference with GrimAA (MV-IVW β: 0.58, 95% CI: 0.27–0.88, *p <* 0.001), and PhenoAA (MV-IVW β: 0.68, 95% CI: 0.28–1.08, *p =* 0.001). Regarding body fat percentage, associations with increased GrimAA (MV-IVW β: 0.76, 95% CI: 0.42–1.09, *p <* 0.001) and HannumAA (MV-IVW β: 0.32, 95% CI: 0.00-0.65, *p =* 0.047) were observed after adjusting for dentures (Fig. 3). The robustness of the MV-IVW estimates was confirmed by at least MVMR Egger sensitivity analyses (Supplementary Table 11). MR Egger intercept estimates suggested no pleiotropy in all analyses. The other potential mediators (CRP and health satisfaction) were excluded because they were not causally associated with EAA (Supplementary Table 10).

### Mediating effects of mediators in the associations between dentures and EAA

**Fig. 4:**
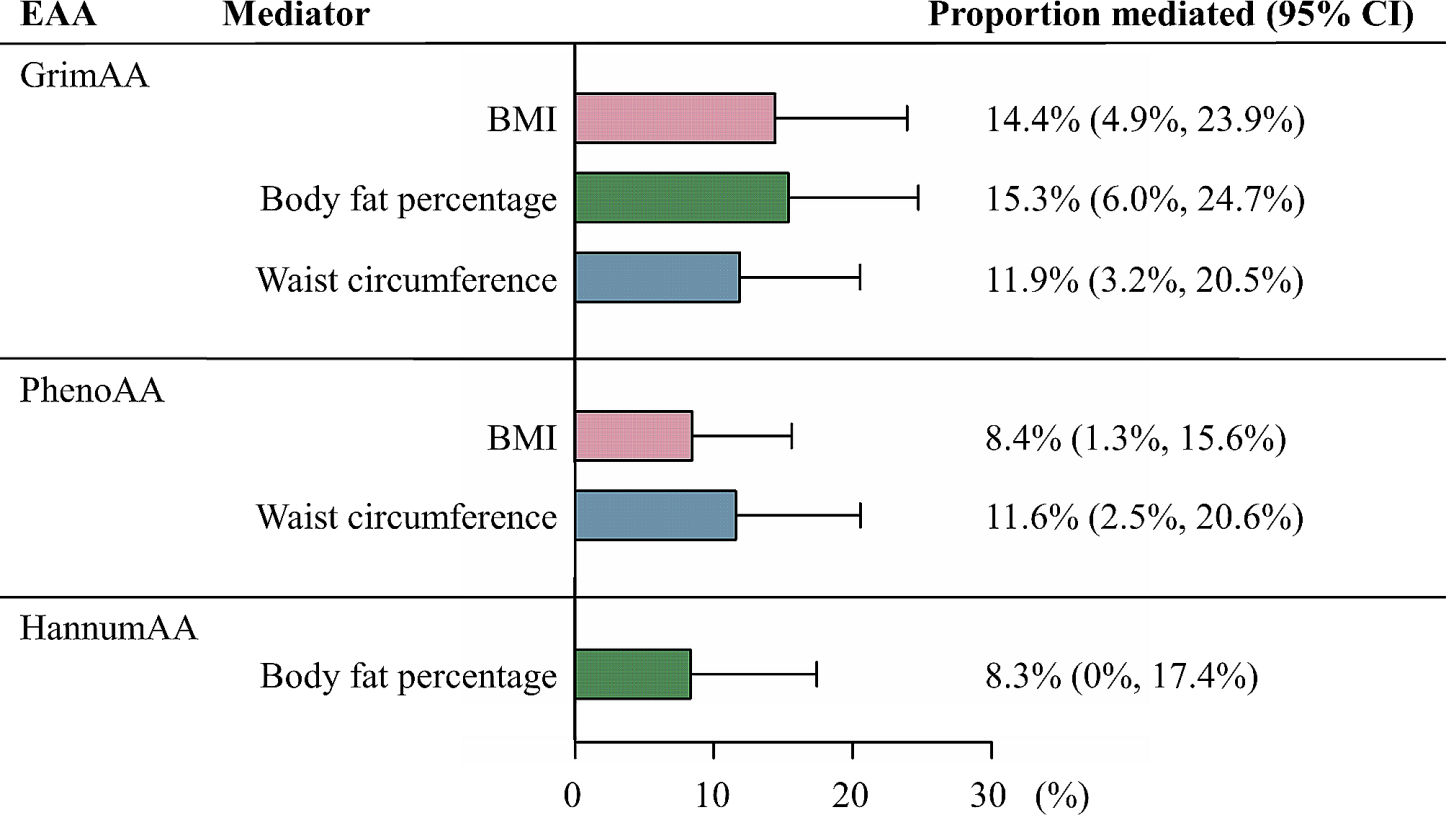
Two-step MR estimating proportions mediated by mediators in the causal associations of dentures with epigenetic-age acceleration. Histograms (bars) represent the mediation proportions (95% CI). Pink plots represent the proportions mediated by BMI, green plots represent the proportion mediated by body fat percentage, blue plots represent the proportion mediated by waist circumference. CI, confidence interval. EAA, epigenetic-age acceleration; GrimAA, GrimAge acceleration; PhenoAA, PhenoAge acceleration; HannumAA, HannumAge acceleration; BMI, body mass index

Ranked by mediation proportion, body fat percentage mediated 15.3% of the total effect of dentures on GrimAA, followed by BMI (mediation proportion: 14.4%, 95% CI: 4.9-23.9%) and waist circumference (mediation proportion: 11.9%, 95% CI: 3.2-20.5%) (Fig. 4). In the case of PhenoAA, the primary mediators by proportion were waist circumference (mediation proportion: 11.6%, 95% CI: 2.5-20.6%) and BMI (mediation proportion: 8.4%, 95% CI: 1.3-15.6%). Notably, only body fat percentage played a mediating role, contributing 8.3% to the total effect of dentures on HannumAA.

## Discussion

To date, this study marks the initial attempt to explore the causal connections between dental traits and EAA, employing various complementary MR methodologies. Our investigation has yielded unique findings, indicating that the genetic inclination towards denture usage has a causal effect on heightened GrimAA, PhenoAA, and HannumAA, even after adjustment for periodontitis, the number of teeth, bleeding gums, and caries. Further analyses proposed that this discerned causation might be driven by obesity-related traits.

A numerous of epidemiologic evidence has currently emerged, revealing a positive relationship between self-reported or clinically measured oral health issues, including periodontitis, dentures, edentulous conditions, and caries, with the risks of frailty and shortened telomeres [26–28]. Telomere length and EAA indicate distinct mechanisms in the aging, each independently linked to the risk of mortality and chronological age [1]. Our findings contribute novel evidence underscoring the importance of dental health in influencing EAA. On the contrary, some argued that improved masticatory function due to denture wearing could diversity nutrition, food intake, and selection, thereby promoting better and longer life. For example, Shimazaki et al. discovered that edentulous subjects without dentures encountered a notably increased mortality rate, irrespective of their physical and mental health status at the baseline [29]. Findings from available cross-sectional studies confirm a negative association between denture wearing and mortality rates [30–32]. Specifically, in a 15-year follow-up study involving 5,688 participants, it was observed that dentures only played a protective role in subjects with fewer than 10 functional teeth [33]. It is crucial to acknowledge that these findings might be constrained by several factors, including the lack of healthy controls, insufficient sample sizes, limited information on new denture wearers during follow-up, and uncontrolled covariates. Additionally, the advanced age of the covered population (often > 80 years) might bias the results, considering the target audience for dentures.

Up to data, the most extensive prospective study in this field hails from the Korea Longitudinal Study of Aging (2006–2018), encompassing a sizable cohort of 10,254 adults who underwent meticulous dental examination conducted by trained investigators [27]. The subsequent analysis showed denture users exhibited a heightened prevalence of frailty or mortality. Multivariate logistic regression analyses additionally indicated denture wearing as a compelling risk factor for aging within the population. This study holds more credibility due to its substantial sample size and the adjustments made for critical variables, including chewing ability, age, common chronic diseases, daily exercise, and socioeconomic status. Moreover, this survey covered participants from diverse age groups, beginning at 45 years. Among a limited number of studies investigating potential causality, Shungin et al. drew attention to a genetic link between denture use/DMFS and earlier parental death―a robust indicator of individual aging [16]. The authors posited that wearing dentures, such as removable partial dentures (RPDs), could impede the flow of food and hinder the self-cleaning action of the buccal mucosa and tongue, ultimately resulting in the buildup of dental plaque on the prosthesis and the surrounding tissue [11]. Notably, 65% of denture plaque biofilms are colonized by recognized respiratory pathogens, including *Streptococcus aureus*, *Pseudomonas aeruginosa*, and *Haemophilus influenzae B* [11]. In particular, *Candida albicans* have the potential to promote the growth of respiratory pathogens and escalate the frequency of chronic obstructive pulmonary disease (COPD) exacerbations [34]. Based on these theories and pertinent observational studies, researchers have propounded the concept of the “oral microbiota-COPD-longevity” axis, underscoring the profound impact of oral bacterial on the EAA [35]. Beyond microbial dysbiosis, the potential influence of dentures on pro-inflammatory signaling through prolonged exposure to toxic particles is conceivable [36]. Despite the general biocompatibility of acrylic resin materials, evidence suggested that unpolymerized components and by-products of the polymerization reaction may have deleterious effects [37].

Our study extends the literature on the impact of dentures in aging in several dimensions. Firstly, within the MR framework, our findings offer robust evidence supporting the causal link between genetic predisposition for denture using and heightened EAA except for IEAA. This observation could imply heterogeneity in the pathology underlying IEAA compared to the other three EAA indicators. Among these, GrimAA outperforms previously available DNA-methylation-based predictors when it comes to predicting death [22]. In particular, the causal inference in this study found support in the consistent magnitude and direction of effects estimated across various MR methods. Even when controlling for number of teeth, caries, periodontitis, and bleeding gums in MVMR analyses, genetic predisposition for denture wearing maintained a strong association with GrimAA, PhenoAA and HannumAA. This suggests that potential bias related to periodontitis and caries, which might lead individuals to use dentures, is less likely in this study. Secondly, the IVs identified in our study specifically predicted genetic susceptibility to denture use, without associations with denture stomatitis, denture misuse, or unreasonable design, implying that potential health risks of dentures should be considered even following proper use. Notably, the MVMR analyses additionally revealed that the causality of genetic susceptibility to dentures on EAA was mediated by obesity (e.g., BMI, body fat percentage, and waist circumference). These finding emphasize that the underlying biological conditions associated with genetic variants of dentures might contribute to the accelerated aging, rather than the prosthesis itself. Clinical utilization of dentures might indicate specific health conditions that could lead to aging-related disorders. In this study, we determined that genetic predisposition to dentures causally related with obesity and CRP, established risk factors for EAA [1, 14]. Interestingly, further MVMR analysis suggested that it was obesity, not CRP, that played a mediating role in the impact of dentures on EAA. In general, dentures are regarded as the final stage of oral health issues and may serve as an indicator of edentulous dentition. In the UK, self-reported dentures are likely to refer to removable dentures. Based on the Adult Dental Survey conducted in 2009/2010, a total of 20% of the UK population utilized some type of removable dentures and demonstrated an increased incidence of obesity [38]. Individuals wearing dentures often exhibit a tendency to choose processed foods that are high in fat and sugar. This dietary pattern can result in an insufficient intake of vitamins and minerals, attributed to the challenges and fatigue associated with chewing [39]. In addition, mood disturbances, particularly depression, are common secondary symptoms of edentulous dentition or dentures wearing, further linking oral health to obesity [40, 41]. Given that most denture wearers are elderly individuals with numerous age-related factors such as obesity and larger waist circumferences, confounding by these conditions must be acknowledged. However, our two-step MR approach also yielded results for denture effects on EAA after adjusting for obesity-related parameters (Supplementary Table 11). Specifically, dentures maintained a significant causal relationship with GrimAA after adjusting for BMI, body fat percentage, and waist circumference (MV-IVW *p* ≤ 0.013). For HannAA, the causal effect of dentures remained significant after adjusting for body fat percentage or waist circumference (MV-IVW *p* ≤ 0.045). Genetic predisposition to denture use also maintained a causal relationship with PhenoAA after adjusting for body fat percentage (MV-IVW *p* = 0.011). Thus, the observed causal effects in our study remained robust even when considering obesity.

However, these obesity-related factors alone may not fully account for the observed relationship. Other potential mediators warrant consideration. For instance, dietary habits characterized by high consumption of processed foods, sugar, and saturated fats, and low intake of fruits, vegetables, and whole grains, can disrupt trace element homeostasis, such as iron levels, contributing to anemia and accelerating epigenetic aging in denture wearers [10, 42]. Chronic stress associated with denture wear may dysregulate the hypothalamic-pituitary-adrenal axis, elevating cortisol levels [43]. Prolonged exposure to high cortisol levels can accelerate biological aging by promoting inflammation, oxidative stress, and telomere shortening [44]. Moreover, denture wearers experiencing dissatisfaction or social stigma related to their dental prostheses may be more susceptible to psychological disorders, further accelerating biological aging [45].

Utilizing a multidisciplinary strategy has proven remarkable efficacy in addressing EAA. Our findings provide new evidence and strategies for aging and dental health interventions. The study highlights denture wearers as a potentially high-risk group for EAA, emphasizing the importance of routine physical examinations and preventive measures such as regular dental check-ups, prompt treatment of dental issues, and oral hygiene education to reduce the need for dentures and their impact on aging. Furthermore, heightened attention to obesity in denture wearers, focusing on monitoring and managing obesity-related indices, is crucial for reducing biological aging and mortality in this population. Clinicians may recommend weight management interventions such as dietary adjustments and increased physical activity. However, recent research among denture wearers revealed concerning statistics: 54.10% of the participants reported the absence of information on denture care and 19.17% wore denture overnight [46]. Prosthodontist, especially, have the potential to significantly influence the current and future well-being of denture wearers through active dental education and weight evaluation and management. Collaboration among dental professionals, nutrition experts, and other healthcare practitioners can guarantee comprehensive care and tackle the intricate interconnection between dentures and EAA.

It is important to note that the use of dentures significantly improves masticatory function, allowing for a wider variety of foods and better nutrition [27]. Additionally, dentures enhance phonetics, improving speech clarity and thereby enhancing social interactions and self-confidence. Aesthetically, dentures restore a natural appearance, boosting self-esteem and enhancing quality of life. By investigating various aspects of denture use and their potential impacts on health outcomes, we aimed to provide valuable insights that can inform clinical practice and public health initiatives. Ultimately, our goal is to contribute to the improvement of health outcomes and the overall well-being of individuals who wear dentures.

However, it is essential to be cautious when interpreting the findings of the current investigation, given the presence of several limitations that necessitate consideration. Firstly, this examination was exclusively carried out within a European demographic, posing challenges to the extrapolation of results due to potential impacts of diverse dietary composition and oral-health education on causality. Secondly, MR investigations evaluate lifelong associations, rendering the magnitude of the effect not directly comparable to short-term intervention studies. Thirdly, despite adjusting the SNPs used in the UVMR analysis for diabetes, smoking, and alcohol consumption, other notable confounders such as socioeconomic status (SES), diet, and nutrition may not have been thoroughly considered. The causal relationship could be biased, either overestimated or underestimated, due to the influence of these unmeasured confounders [47]. Higher SES is often linked with better access to healthcare, including dental care, and improved health literacy, which can influence both oral health and overall aging. Consequently, the failure to adjust for SES might lead to an overestimation of the impact of dental traits on EAA. Additionally, the observed association might be spurious, attributing effects to dental traits that could actually be due to differences in SES or nutritional status, thus compromising the internal validity of our study. Such limitations also affect the generalizability of our findings, as relationships observed in one population might not hold true in others with different SES or nutritional profiles. Misidentifying the causes of observed relationships can hinder effective public health interventions. To mitigate these issues, future analyses should incorporate SES, nutrition, and other relevant confounders into statistical models, conduct stratified analyses, and perform sensitivity analyses to ensure robust findings. Fourthly, advancements in DNA-methylation-based age predictors have emerged since the initiation of these EAA analyses. In comparison to the original Hannum and Horvath clocks, the Zhang clock demonstrates less sensitive to cellular heterogeneity [48]. However, no such GWAS data are available. Fifthly, information detailing the types of dentures (e.g., RPD, dental implants, and fixed bridges) and the duration of denture wearing was unavailable, potentially limiting data interpretation, though potential impacts could be alleviated through MR analysis [38]. Further studies with detailed evaluations for denture use or other oral status are needed.

## Conclusion

Our MR estimates furnish compelling evidence regarding the causal effects of genetic predisposition to denture wearing on increased GrimAA, PhenoAA, and HannumAA, potentially mediated by obesity-related traits. These findings offer novel perspectives on the mechanisms underlying the relationship between dentures utilization and EAA and may suggest to healthcare professionals the importance of enhanced focus on monitoring and managing obesity to decelerate the EAA in denture wearers. Longitudinal studies are essential to assess the long-term impacts of dental traits such as dentures or tooth implants on biological aging markers. Future research could explore specific molecular pathways linking dental health to biological aging, including inflammatory markers, oxidative stress pathways, and epigenetic modifications in denture wearers. Understanding these mechanisms could inform targeted interventions aimed at mitigating the impact of dental health on biological aging, thereby enhancing clinical outcomes and public health strategies.

### Electronic supplementary material

Below is the link to the electronic supplementary material.

Supplementary Material 1

## Data Availability

Data is provided within the manuscript or supplementary information files.
